# Role of endoscopic evaluation prior to diagnostic transesophageal echocardiography: Is it necessary?

**DOI:** 10.1002/jgh3.12792

**Published:** 2022-07-19

**Authors:** Sunny Sandhu, Dhuha Alhankawi, Marina Roytman, Ratnali Jain, Devang Prajapati

**Affiliations:** ^1^ Department of Internal Medicine University of California, San Francisco – Fresno Fresno California USA; ^2^ Department of Gastroenterology and Hepatology University of California, San Francisco – Fresno Fresno California USA; ^3^ Clinical Research Center University of California, San Francisco – Fresno Fresno California USA; ^4^ Department of Gastroenterology and Hepatology VA Central California Healthcare System Fresno California USA

**Keywords:** esophageal varices, esophagogastroduodenoscopy, stricture, transesophageal echocardiography

## Abstract

**Background and Aim:**

Esophagogastroduodenoscopy (EGD) is often performed prior to transesophageal echocardiogram (TEE) to evaluate for esophageal pathologies. Although TEE is a safe procedure, some contraindications exist, such as esophageal varices. The incidence of bleeding with TEE is <0.01%, which questions the need for this routine invasive procedure prior to TEE. We sought to characterize patients in whom pre‐TEE endoscopy was requested to determine its clinical utility and identify those that would most benefit.

**Methods:**

We retrospectively studied patients who underwent EGD for TEE clearance between January 2014 and October 2019. We assessed how often EGD changed management and complications after TEE in those with EGD abnormalities.

**Results:**

Eighty‐three patients were included. Twenty‐three percent had prior GI bleed, 63% had cirrhosis, 18% had known varices, and 7% had prior variceal bleed. The most common EGD findings were varices (33%). Eighty‐one percent proceeded with TEE. Reasons for TEE deferral included varices (12.5%), high‐risk bleeding lesion (12.5%), and mechanical abnormality (12.5%). In the majority (37.5%), TEE was deemed no longer indicated. No patient undergoing TEE had significant hemoglobin drop or overt bleeding. The most common reason for not performing TEE was unrelated to EGD findings: lack of ongoing indication for TEE.

**Conclusion:**

Based on our study, EGD is likely not needed for TEE clearance in patients with varices or prior GI bleed. Given that data are limited in patients with abnormalities such as strictures, EGD may still be warranted for these patients. Further studies to identify which patients will benefit from pre‐TEE endoscopy are warranted.

## Graphical abstract

Esophagogastroduodenoscopy (EGD) is often performed prior to transesophageal echocardiogram (TEE) to evaluate for esophageal pathologies. Based on our study, EGD is likely not needed for TEE clearance in patients with varices or prior GI bleed.

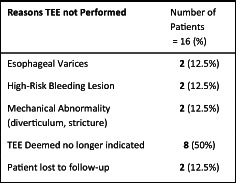

## Introduction

Transesophageal echocardiogram (TEE) is a fairly common procedure performed both in the ambulatory and intraoperative setting.^1^, ^2^, ^3^ It is largely regarded as a safe procedure; however, several contraindications and relative contraindications do exist, and not infrequently do echocardiographers require additional studies to be done prior to performing TEE.^4^, ^5^ Contraindications include an array of esophageal structural pathologies including strictures, diverticula, varices, or masses.^6^ Associated concerns can range from potential anatomic challenges, bleeding, to esophageal perforation.^1^ However, supporting data regarding the safety of TEE and complication rates with specific esophageal abnormalities are extremely limited. For this reason, gastroenterologists are often consulted for urgent inpatient endoscopic evaluation with esophagogastroduodenoscopy (EGD) prior to TEE. The reasoning for this theoretical risk of complications when inserting the TEE probe, which lacks direct visualization.^7^

Historically, one of the most common reasons for seeking clearance has been concern for underlying esophageal varices. However, there has been no consistent data to suggest variceal‐related complications from performing TEE. Recent data have suggested that patients who undergo TEE actually have an extremely low incidence of bleeding.^3^, ^7^, ^8^, ^9^, ^10^, ^11^, ^12^, ^13^, ^14^, ^15^, ^16^, ^17^, ^18^ The incidence of major bleeding with diagnostic TEE has been reported to occur in less than 0.01% of cases.^4^ This has led some to question the significance of the theoretical risk of mechanical trauma. In regards to current practice guidelines, the American Society of Anesthesiologists has an equivocal view on whether esophageal varices should be a contraindication to TEE.^16^ Conversely, other groups such as the American Society of Echocardiography and the Society of Cardiovascular Anesthesiologists list the presence of esophageal varices as a relative contraindication to TEE, and recommend evaluation of patients on a case‐by‐case basis and consideration of clearance by a gastroenterologist prior to pursuing TEE.^4^ As a result, depending on the comfort level of local echocardiographers in the United States, gastroenterologists may commonly be consulted for EGD on patients prior to TEE for “clearance.” It is important to also note however that there are no current recommendations or guidelines that exist for gastroenterologists on how to sufficiently “clear” these patients to proceed with TEE.^7^

Overall, the practical benefits of performing EGD prior to TEE have not yet been fully elucidated.^11^ In our literature search, there were little data available on how many patients actually undergo EGD prior to TEE for clearance, and how frequently in those cases it has been beneficial in changing management moving forward. We found only one study that evaluated if upper endoscopy prior to transesophageal echocardiography changed patient management. Zuchelli *et al*. performed a retrospective analysis at an inner‐city tertiary care center, which included 134 patients who underwent an EGD to evaluate the safety prior to blind passage of a TEE probe. Of the 134 patients, 20 were not cleared for TEE, 17 of whom had “esophageal structure abnormalities” including esophageal varices, stricture, ring, web, or Zenker diverticulum. Given that 15% of EGDs done prior to TEE changed management in their limited population, they concluded that EGD may possibly be clinically beneficial before TEE.^17^

At present, the ambiguity of the current guidelines and wide variation in practice for performing TEE may lead to a large number of patients undergoing an additional invasive procedure that may actually be unnecessary. Given that the data available from other single‐center studies are fairly limited, we sought to characterize the patients in whom pre‐TEE endoscopy was requested to determine its clinical utility and possibly identify those who would most benefit from this intervention.

## Materials and methods

We retrospectively studied patients who underwent EGD for TEE clearance at Community Regional Medical Center (CRMC) between January 2014 and October 2019. Our study was approved by our local institutional review board (IRB), and all data were stored on a secure server. Patients <18 years of age were excluded. Gastroenterology consults during this period were reviewed in Epic, our institution's electronic medical record system. The data reviewed included baseline demographics (Table 1) such as age, gender, BMI, and race (Hispanic, Caucasian, African American, or Other). Social history was reviewed to determine if the patient had a history of past or present alcohol abuse, which we defined as drinks >3/day or >14/week for men, and >2/day or >7/week for women. We also reviewed past medical history for any history of known cirrhosis, etiology of cirrhosis, history of known esophageal abnormalities, grade of existing varices if known, prior endoscopic or pharmacologic treatments for varices, and history of any prior GI bleed. We included patients who were referred for EGD for TEE clearance, and the listed reasons for the requests for EGD were due to a history of specific complaints, which were not ongoing or active issues. In patients who had a suspected upper GI bleed, most of the patients had a history suggestive of possible GI bleed due to clinical concerns including a history of melena or unexplained anemia, which was currently not active. One patient had a history of coffee‐ground emesis, which had since resolved. None had signs of clinical instability or an overt upper GI bleed. We reviewed endoscopy reports, laboratory values, progress notes, and TEE procedure reports. We documented the number of EGDs performed at our center for TEE clearance as well as how often subsequent TEE was deferred due to EGD findings. We documented the endoscopic abnormalities in patients who were not cleared for TEE. We also reviewed if complications occurred after TEE, and defined bleeding events as hematemesis, melena, hematochezia, pRBC transfusion, or hemoglobin decrease ≥2 g/dL from baseline within 48 h after the procedure. Basic frequencies were run and percentages were calculated using SPSS version 27.

**Table 1:** Patient characteristics/past medical history

	Total number of patients = 83 (%)
Gender	
Male	56 (67%)
Female	27 (33%)
Ethnicity	
Caucasian	28 (34%)
African American	1 (1%)
Hispanic	49 (59%)
Other	5 (6%)
History of alcohol abuse	48 (58%)
Diagnosis of cirrhosis (alcoholic = 30, hepatitis C = 19, non‐alcoholic fatty liver disease = 1, other = 2)	52 (63%)
History of GI bleed (varices = 6, peptic ulcer = 3, esophagitis = 3, gastritis = 1, arteriovenous malformation = 1, other = 1, unknown = 1)	19 (23%)
History of esophageal abnormalities	26 (31%)
Esophagitis	6 (7%)
Stricture	4 (5%)
Diverticulum	1 (1%)
Esophageal varices	15 (18%)
Small varices	9 (11%)
Large varices	6 (7%)

## Results

A total of 83 patients met our inclusion criteria to be included in our study. The most common reasons for EGD prior to TEE were a clinical suspicion for portal hypertension in 43% (36/83) of patients and suspected upper GI bleed in 20% (17 of 83) of patients. Dysphagia was the third most common reason for EGD prior to TEE, accounting for 18% (15 of 83) of cases (Table 2). The most common indication for TEE was evaluation for infective endocarditis in 72% (60 of 83), followed by cardiac surgery in 27% (22 of 83).

**Table 2:** Reasons for endoscopy request

Reasons for endoscopy	Number of patients = 83 (%)
Known varices on prior EGD	14 (17%)
Clinical suspicion for portal hypertension	36 (43%)
Dysphagia	15 (18%)
History of true/suspected upper GI bleed	17 (21%)
Other	1 (1%)

A total of 23% (19 of 83) of patients had a history of prior upper GI bleed from various etiologies. In regards to past medical history, 63% (52 of 83) of patients had a known history of cirrhosis, of which 29% (15 of 52) of patients had documented history of esophageal varices, and 12% (6 of 52) had a prior variceal hemorrhage. With respect to social history, most patients had a history of alcohol abuse, which was present in 58% (48 of 83) of cases.

Of the 83 patients who underwent EGD, the most common findings were esophageal varices, present in 33% (28 of 83) of endoscopies. Sixty‐eight percent (19 of 28) of esophageal varices were small varices and 32% (9 of 28) were large varices. The next most common EGD findings were normal findings in 29% (24 of 83) and esophagitis in 24% (20 of 83). Diverticulum and stricture, which have traditionally been considered absolute contraindications for TEE, were present in 6% (5 of 83) of cases (Table 3). Recommendations by the gastroenterologist for the safety of TEE were not provided in 87% of cases. Only one patient had a recommendation against TEE as EGD showed an esophageal stricture requiring dilation. In total, 81% (67 of 83) of all patients subsequently proceeded with TEE. Of those who did not undergo TEE, the most common reason was due to being deemed no longer indicated in 50% (8 of 16) of cases, and an additional 12.5% (2 of 16) patients were actually lost to follow‐up (Table 4). Only 7% (6 of 83) of patients had a deferral of TEE. The most common reasons for TEE deferral were esophageal varices in 12.5% (2 of 16), high‐risk bleeding lesions in 12.5% (2 of 16), and mechanical esophageal abnormality in 12.5% (2 of 16). Of the 67 patients in our study that proceeded with TEE, 33% (22 of 67) had esophageal varices (15 had small varices, 7 had large varices), 4% (3 of 67) had a stricture. No complications occurred from TEE in our study. Of the 67 patients who proceeded with TEE, none had a significant hemoglobin drop (defined as >2 g/dL), clinically observed overt gastrointestinal bleeding, or perforation. None of the patients had a technically difficult TEE attributed to the endoscopic findings.

**Table 3:** EGD findings

EGD findings	Number of patients = 83
Normal	24
Small varices	19
Large varices	9
Esophagitis	20
Diverticulum	1
Stricture	4
Other	6

**Table 4:** Reasons TEE not performed

Reasons TEE not performed	Number of patients = 16 (%)
Esophageal varices	2 (12.5%)
High‐risk bleeding lesion	2 (12.5%)
Mechanical abnormality (diverticulum, stricture)	2 (12.5%)
TEE deemed no longer indicated	8 (50%)
Patient lost to follow‐up	2 (12.5%)

Subgroup analysis of the 83 patients who underwent EGD was also performed. Of the 18 patients who had a suspected upper GI bleed as a reason for EGD prior to TEE, none had endoscopic findings that precluded subsequent TEE, and only one of these patients did not proceed with TEE as it was deemed no longer indicated. Additionally, only 8.3% (3 of 36) of those who had a clinical suspicion for portal hypertension had EGD findings that precluded TEE. In comparison, 13% (2 of 15) patients who reported a history of prior dysphagia as a reason for EGD prior to TEE had an abnormality that precluded TEE. One of these patients had a stricture that required dilation, and the other had findings concerning esophageal dysmotility.

When comparing cirrhotic to non‐cirrhotic patients, it was found that about 7.7% (4 of 52) of patients with cirrhosis had EGD findings that precluded TEE, compared with about 6% (2 of 31) in non‐cirrhotic patients. Both of these non‐cirrhotic patients had a history of dysphagia as a reason for EGD prior to TEE.

## Discussion

This study of 83 patients from CRMC is among the largest that has been performed. Based on our study, we propose that EGD is likely not needed specifically for TEE clearance. In our group of patients, EGD performed for TEE clearance changed subsequent management in only 7% (6 of 83) of cases. This is much lower than the 15% observed in the study performed by Zuchelli *et al*., which shows that current practices likely differ widely throughout institutions.^17^ This calls into question the overall utility as it did not change management significantly. Interestingly, the most common reason in our study for not proceeding with TEE was unrelated to EGD findings—it was due to the lack of ongoing indication for TEE. Although some of the patients in our study would have certainly qualified for an elective outpatient EGD if the reasons that were cited were active issues, none of the patients had active concerns, and all of these patients were actually referred for TEE clearance only. The listed reasons were all cited as reasons for performing a TEE due to potential complications associated with TEE specifically. There were no indications in our study that warranted an emergent or urgent inpatient EGD in these patients, including patients having concerns for an active GI bleed.

Although most patients undergoing EGD had cirrhosis, there was not a significant difference in management when compared with patients without cirrhosis. Additionally, there were no complications in any of the patients with cirrhosis who underwent TEE. Although 33% of patients who proceeded with TEE had varices, none of these patients had bleeding complications from TEE. Our data support other small studies, which suggest that TEE may actually be very safe in cirrhotic patients with known varices. Although the presence or concern for cirrhosis can prompt a gastroenterology consult for EGD prior to TEE, our data show that subsequent management did not change significantly in cirrhotic patients vs. non‐cirrhotic patients despite upper endoscopy findings. Additionally, all of the patients with concerns for upper GI bleed subsequently proceeded with TEE, which suggests that EGD may not be required specifically prior to TEE in patients without cirrhosis as well. These findings raise the concern that many medical centers may be unnecessarily subjecting patients to an invasive procedure, which can potentially even delay more appropriate care.

Importantly, although dysphagia as a reason for EGD prior to TEE only represented 15 of 83 patients in our study, 2 of 15 of these patients had underlying esophageal abnormalities that precluded TEE. Given the potential for underlying anatomic pathologies such as strictures or diverticulum in patients with dysphagia, EGD may still be warranted for these patients. As only 15 patients were included with dysphagia in our study, this likely represents a small sample size. Further studies to elucidate the benefits of pre‐TEE endoscopy, specifically in patients with dysphagia, are warranted. Although EGD should be performed in there are active clinical concerns or indications, the indications for EGD referral in our study were primarily for TEE clearance, as there were specific concerns of complications and overall safety of TEE in the setting of potential underlying upper GI pathology. The concerns were listed as the reasons for request for EGD by the cardiology department, which was performed in a more urgent setting during inpatient admission prior to TEE. Although performing a TEE is certainly at the discretion of the cardiologist, due to a lack of guidelines, gastroenterologists are consulted at many centers throughout the United States to perform an EGD for TEE clearance. As current data are limited in the clinical utility of this procedure being performed on an urgent inpatient basis, there are currently no guidelines for both specialists to refer to, which leads to EGD being performed urgently during inpatient admission prior to TEE. Performing EGD in our study did not change the decision to perform TEE, calling into question the need for this in a significant number of cases. In future separate studies, a control group would also be interesting to compare the safety and outcome data of those who had similar reasons for TEE clearance, but were not referred for TEE. We were unfortunately unable to include any patients who were not referred for an EGD, as we were only able to access patients with a referral to GI in our study.
